# Molecular characterization and reference mitogenome of the hookworm *Uncinaria criniformis* (Goeze, 1782) from the Eurasian badger

**DOI:** 10.1017/S0031182025100760

**Published:** 2025-09

**Authors:** Georgiana Deak, Jan Šlapeta

**Affiliations:** 1 Department of Parasitology and Parasitic Diseases, University of Agricultural Sciences and Veterinary Medicine of Cluj-Napoca, Cluj-Napoca, Romania; 2 Sydney School of Veterinary Science, Faculty of Science, The University of Sydney, Sydney, NSW, Australia; 3 Sydney Institute for Infectious Diseases, The University of Sydney, Sydney, NSW, Australia

**Keywords:** Ancylostomatidae, *Dochmoides*, ITS rDNA, *Meles*, mitochondrial genome, mtDNA, phylogenetics

## Abstract

Hookworms are common parasites of Eurasian badgers (*Meles meles*), typically identified as *Uncinaria criniformis*. The taxonomic distinction from *Uncinaria stenocephala*, a species found in dogs and foxes, has long been debated. In this study, we molecularly characterized *U. criniformis* from a Eurasian badger in Romania using genome skimming. We assembled the complete mitochondrial genome and internal transcribed spacer (ITS) rDNA region from 2 adult hookworms morphologically consistent with *U. criniformis*. Phylogenetic analysis of 12 mitochondrial protein-coding genes demonstrated strongly supported clade of *U. criniformis* with *Ancylostoma* spp. ITS rDNA and *cox*1 sequence comparisons revealed only 92.4–92.8% and 88.0–88.5% identity, respectively, between *U. criniformis* and *U. stenocephala*, confirming their molecular distinctiveness. In contrast, our sequences showed >99% identity to sequences from *Arthrostoma leucurus*, a hookworm recently described from the Asian badger (*Meles leucurus*), suggesting conspecificity. These findings support the validity of *U. criniformis* as a distinct species parasitizing *M. meles*, and we propose *A. leucurus* as a junior synonym of *U. criniformis*. Our results highlight the polyphyly of the genus *Uncinaria* and point to the need for broader mitogenomic sampling of hookworms. The molecular markers generated here provide a reference for future parasitological surveys and wildlife disease studies.

## Graphical abstract

**Figure:**
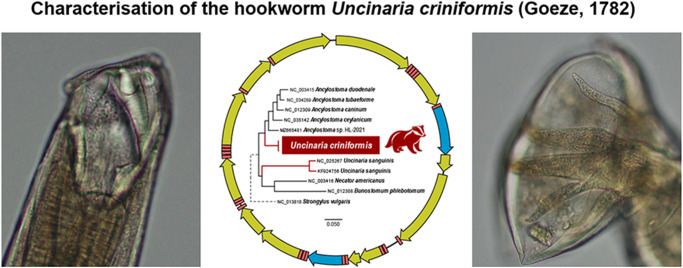

## Introduction

The badger hookworm was first observed and described by the German zoologist Johann August Ephraim Goeze (1731–1793), who mentioned it in his 1782 monograph ‘*Versuch einer Naturgeschichte der Eingeweidewürmer thierischer Körper.’* Goeze (1782) described worms found at the terminal end of the intestine of the Eurasian badger, originally referred to as *Ursus meles* and now classified as *Meles meles* (Linnaeus, 1758). He named the worms *Ascaris criniformis* and used the term ‘uncinate’ to describe their shape. The Latin word *uncinatus* means ‘hooked’ or ‘having a hooked shape’, and the accompanying illustrations of both male and female worms reflect this morphology. Notably, the female worm appears to possess buccal capsule with a characteristic form that would today be recognized as typical of hookworms. In 1798, Josef Aloys Frölich (1766–1841) reviewed intestinal worms and introduced the genus *Uncinaria* for hookworms. The first species Frölich (1789) assigned to this genus was *Uncinaria melis*, from the Eurasian badger, which he considered synonymous with *Ascaris criniformis* (Goeze, 1782), and *Strongylus melis* Müller, 1787. According to the principle of priority in zoological nomenclature, the earliest valid name must be used; therefore, *Uncinaria criniformis* (Goeze, 1782) is the valid name. In 1809, Karl Asmund Rudolphi (1771–1832), a Swedish-born German naturalist often regarded as the father of helminthology, referenced the badger hookworm in Volume II of his *Entozoorum, sive vermium intestinalium: historia naturalis*, under the name *Strongylus criniformis*. Rudolphi (1809) included a comprehensive list of synonyms, such as *U. melis* (Frölich, 1789), and *Ascaris criniformis* (Goeze, 1782). However, Goeze’s original description was considered sparse, and later authors found it too ambiguous to definitively identify the species. Looss (1905) criticized Frölich’s description of *Uncinaria* as inadequate for recognizing the type species of the genus. Similarly, Railliet (1900) referred to Frölich’s *Uncinaria* species as ‘formes spécifiquement indeterminables’ (species of indeterminable specificity).

Morphological comparisons of hookworms from Eurasian badgers with those from domestic dogs and foxes have generated ongoing controversy (Cameron, 1924; Fülleborn, 1924; Ransom, 1924). Over time, the debate has narrowed to focus on whether 2 species are distinct orconspecific: (1) *U. criniformis* (Goeze, 1782) from the Eurasian badger, and (2) *U. stenocephala* (Railliet, 1884) from dogs and foxes (Baylis, 1933; Wolfgang, 1956). In addition to questions of species identity, the genus name has also been a source of debate. Cameron (1924) proposed abandoning the name *Uncinaria* in favour of a new genus, *Dochmoides*, for these hookworms. However, the use of *Dochmoides* has largely fallen out of favour in more recent literature. The name and species *U. criniformis* continue to be used in contemporary studies of Eurasian badger parasites (Seguel and Gottdenker, 2017; Byrne et al., 2020). To date, no molecular markers have been generated for *U. criniformis*, and thus no molecular comparisons with other hookworm species have been possible.

Molecular diagnostics for wildlife parasite surveillance, particularly through non-invasive methods such as metabarcoding, are only effective when reference databases are complete (Redman et al., 2019; Ilik et al., 2024). While barcode libraries are now well-developed for veterinary-relevant hosts like ruminants, horses and dogs, they remain incomplete for many wildlife species, despite the availability of morphological records (Cháves-González et al., 2022; Antonopoulos et al., 2024; Mejías-Alpízar et al., 2024; Šlapeta et al., 2024). To enable reliable studies beyond mainstream hosts, it is critical to update and generate molecular barcodes, especially ITS rDNA and *cox*1, from morphologically verified specimens.

The aim of this study was to molecularly characterize *U. criniformis* from the Eurasian badger in order to resolve the current taxonomic ambiguity of this species. This gap was addressed by sequencing and assembling a reference mitochondrial genome and ITS rDNA from hookworms morphologically consistent with the description of *U. criniformis* as provided by Ransom (1924). The availability of these molecular markers enabled us to compare and distinguish *U. criniformis* from other hookworm species.

## Materials and methods

An adult female Eurasian badger (*Meles meles*) was found dead on the roadside in September 2024 in Bihor County, Romania (47°06′37.9″N, 21°50′35.4″E). Collecting road-kills was approved by Ethical Committee Decision no. 232/23.11.2020 (University of Agricultural Sciences and Veterinary Medicine of Cluj-Napoca, Cluj-Napoca, Romania). The intestinal contents were examined for the presence of hookworms. Recovered hookworms were preserved in 70% ethanol (v/v) and transported to the University of Sydney.

Genomic DNA (gDNA) was extracted from individual hookworms after allowing the ethanol to evaporate, using the Monarch Genomic DNA Purification Kit (New England Biolabs, Melbourne, Australia). The extracted gDNA was stored at −20°C.

The gDNA from 2 adult hookworms was used for genome skimming via next-generation sequencing (NGS) on the Illumina NovaSeq 6000 platform, generating 150-bp paired-end reads with approximately 3 Gb of raw sequence data (Novogene, Singapore). The resulting FastQ files were analysed using the Artemis High Performance Computing (HPC) system at the Sydney Informatics Hub, University of Sydney.

The internal transcribed spacer (ITS) rDNA region – comprising the 5′ end of the small subunit ribosomal RNA gene, ITS1, 5.8S rRNA gene, ITS2, and the 3′ end of the large subunit rRNA gene – was assembled from the FastQ data using the MITObim v1.9.1 pipeline (Hahn et al., 2013); https://github.com/chrishah/MITObim, with the *U. stenocephala* sequence (AF194145) as a reference. The assembled ITS rDNA sequences were aligned with available ITS rDNA sequences from other hookworm species using CLC Main Workbench 25.0.1 (CLC bio, Qiagen, Clayton, Australia). ITS2 is a commonly used barcode for nematodes that is used in ‘nemabiome’ studies (Avramenko et al., 2015).

The complete mitochondrial genome (mitogenome) was assembled from the FastQ data using the GetOrganelle v1.7.5.3 pipeline (Jin et al., 2020); https://github.com/Kinggerm/GetOrganelle. The circularized mitogenome was aligned with available complete mitogenomes of other hookworm species using CLC Main Workbench. The mitogenome of *Strongylus vulgaris* was used as the outgroup.

Phylogenetic analysis was based on 12 mitochondrial protein-coding genes, comprising 3,394 aligned amino acid positions. Analyses were conducted in MEGA11 (Tamura et al., 2021). The phylogenetic tree was inferred using the Maximum Likelihood (ML) method with the JTT matrix-based model, incorporating a discrete Gamma distribution to model rate variation among sites (5 categories, + G, parameter = 0.2729), and allowing for a proportion of invariable sites (+I, 35.96%). A second tree was inferred using the Minimum Evolution (ME) method, with distances computed using the JTT model and rate variation modelled with a Gamma distribution (shape parameter = 0.7). Bootstrap analysis was performed to assess node support (1,000 replicates for ME; 100 replicates for ML).

## Results

**Figure 1.**
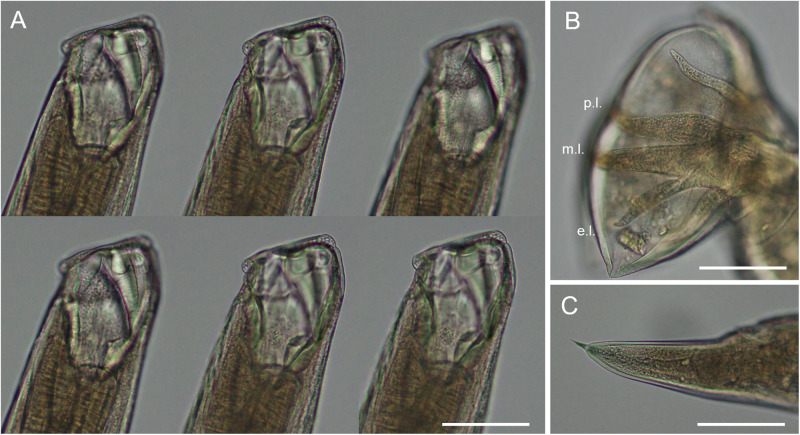
*Uncinaria criniformis* Goetze, 1872 from Eurasian badger (*Meles meles*). (A) buccal capsule in 6 different consecutive lateral focal planes. (B) Male bursa from the lateral view showing the lateral rays (externo-lateral, e.L.; medio-lateral, m.L.; postero-lateral, p.L.). (C) Female caudal end showing a tail with terminal bristle. All images at the same scale, scale bar = 100 µm.

The recovered hookworms measured 6–8 mm in length and closely resembled *U. criniformis* as described by Ransom (1924). In lateral view, the buccal capsule appeared only slightly curved (Figure 1A). The buccal capsule formed an elongate cone with an anterior opening bearing 2 cutting plates and no visible teeth. Despite examining the buccal capsule at multiple focal planes, we were unable to identify distinct articulated plates. Due to ethanol preservation, the relaxation of the specimens was suboptimal, particularly affecting the observation of the male bursa. As a result, the digitation of the dorsal rays could not be determined. However, the lateral rays showed a distinctly narrower externo-lateral ray compared to the medio-lateral and postero-lateral rays, with the medio-lateral ray being slightly thicker than the postero-lateral ray (Figure 1B). Male spicules measured 590–600 µm, and female tails were 130–150 µm long, each ending in a terminal bristle (Figure 1C).

Genomic DNA was extracted from 2 *U. criniformis* specimens (one male and one female), yielding 0.74 ng/μl (JS6867) and 0.49 ng/μl (JS6869). Sequencing of gDNA from voucher JS6867 (GD01-1) produced 27,638,320 raw 150 bp reads, totaling 4.1 Gb of data (Q30 = 91.04%; GC = 42.66%). Voucher JS6869 (GD01-3) yielded 22,037,344 reads, totaling 3.3 Gb (Q30 = 91.01%; GC = 43.43%).

The ITS rDNA region (5′ end of SSU rRNA – ITS1 – 5.8S rRNA – ITS2 – 3′ end of LSU rRNA) was assembled from both FastQ datasets. Each sequence was 757-nt long, differing by 2 mismatches in the ITS1 region. Sequence comparison revealed a close match to 2 ITS rDNA sequences (MN078169 and MK348041), annotated as *Arthrostoma* sp. ex *Meles leucurus* (isolate LK-01-3, China), with 99.7% identity. These sequences differed from our samples by a single indel in ITS2 and one mismatch in ITS1 per sample. In contrast, ITS rDNA sequences from *U. stenocephala* (AF194145, PQ316553, MT345056) showed only 92.4–92.8% identity.

**Figure 2.**
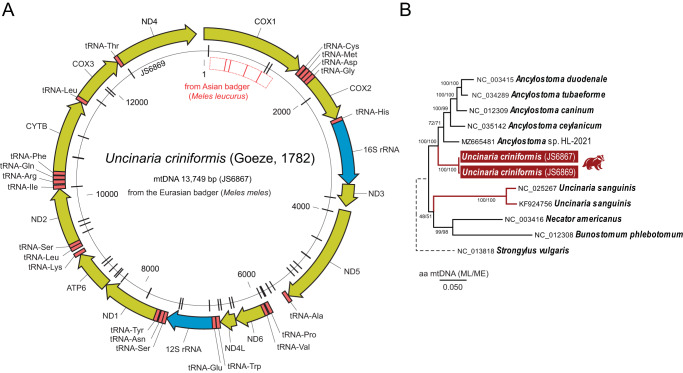
Circular mitogenomes (mtDNA) of two hookworms (*Uncinaria criniformis*) from Eurasian badger (*Meles meles*). (A) all amino acid coding genes are coded by the same DNA strand, transcribed clockwise and labelled by their protein name (yellow). Transfer RNA genes (red) are identified by a 3-letter amino acid code. Two ribosomal RNA genes (rRNA) are indicated in blue. The inner circle consisting of individual radiating lines represents polymorphism between the two genomes (each line represents a single-nucleotide polymorphism). the start of mtDNA is arbitrarily set at the start (1) of the *cox*1 coding for COX1, in addition the positions of 2,000 to 12,000 nucleotides are indicated. The red inner most box within *cox*1 gene region represents hookworm sequence (MW517832) from the Asian badger (*Meles leucurus*) with each bolded line represents polymorphism against *U. criniformis cox*1 sequence. (B) Phylogenetic tree reconstructed using maximum likelihood (ML) method and JTT matrix-based model (log likelihood = −20813.94). the model included gamma distribution (+*G*, parameter = 0.2729) and allowed for some sites to be evolutionarily invariable ([+*I*], 35.96% sites). the tree is drawn to scale, with branch lengths measured in the number of substitutions per site. The percentage of replicate trees in which the associated taxa clustered together in the bootstrap test, 100 replicates for ML and 1,000 for minimum evolution [ME], are shown next to the branches. ME analysis included evolutionary distances computed using the JTT matrix-based method a gamma distribution (shape parameter = 0.7). this analysis involved 12 amino acid sequences and a total of 3,394 positions in the final dataset. Branches with *uncinaria* spp. are drawn in red. *Strongylus vulgaris* mitogenome served as an outgroup.

Complete circular mitochondrial genomes (mitogenomes) were assembled for both specimens: 13,749 nt (JS6867) and 13,748 nt (JS6869). Each mitogenome encoded 12 protein-coding genes (COX1–3, NAD1–6, NAD4L, ATP6, CYTB), 2 rRNA genes, and 22 tRNA genes (Figure 2A). All genes were transcribed in the same direction (5′→3′), with gene boundaries inferred by comparison to related hookworm mitogenomes. The 2 mitogenomes were 99.7% identical, differing by 46 positions, including 3 gaps.

Partial *cox*1 sequences (1,209 nt) showed the closest match to *Arthrostoma* sp. ex *Meles leucurus* (MW517831 and MW517832, isolates M1F and M2M, China), with 99.5–99.8% identity and 3–6 synonymous substitutions. The 2 *U. criniformis* sequences (JS6867 and JS6869) differed by 2 synonymous substitutions (99.8% identity).

Comparison with *U. stenocephala cox1* sequences (PP916662, MW682884, PQ555179) over a 333 nt region revealed only 88.0–88.5% identity, with 38–40 mismatches. In contrast, *U. stenocephala* sequences were 99.1–99.4% identical to each other over the same region.

The newly assembled *U. criniformis* mitogenomes were aligned with available hookworm mitogenomes (*Ancylostoma* spp., *Uncinaria sanguinis, Bunostomum phlebotomum, Necator americanus*). Phylogenetic analysis was based on amino acid sequences of 12 protein-coding genes (3,394 aligned positions). Both Maximum Likelihood (ML) and Minimum Evolution (ME) methods produced identical tree topologies (Figure 2B). *Uncinaria criniformis* formed a strongly supported (100%) monophyletic group with *Ancylostoma* spp. In contrast, *U. sanguinis* formed a polyphyletic group relative to *U. criniformis*. The branching support between *U. criniformis, U. sanguinis*, and the *Necator*–*Bunostomum* clade was weak in both ML (48%) and ME (51%) analyses (Figure 2B).

## Discussion

Hookworm infections in Eurasian badgers (*M. meles*) are commonly reported, with the species typically identified as *U. criniformis* (Magi et al., 1999; Torres et al., 2001; Seguel and Gottdenker, 2017; Byrne et al., 2020). During the early to mid-20th century, taxonomists debated the morphological distinctiveness of *U. stenocephala*, a species mainly found in canids such as dogs and foxes, compared to hookworms found in badgers, which are mustelids (Looss, 1905; Cameron, 1924; Ransom, 1924; Baylis, 1933; Wolfgang, 1956). Although consensus has not been universally reached, *U. criniformis* is now widely used in studies of European wildlife diseases and epidemiology (Seguel and Gottdenker, 2017; Kelly et al., 2022).

To address this taxonomic uncertainty using molecular tools, Górski et al (2023) recently examined hookworms from Eurasian badgers in Poland and concluded that their specimens belonged to *U. stenocephala*, showing molecular identity with *U. stenocephala* from dogs, red foxes, and raccoon dogs. However, this study demonstrates that the ITS rDNA sequence from *U. stenocephala* (OP811914) reported by Górski et al (2023) is clearly distinct from the ITS rDNA sequences obtained here from hookworms identified as *U. criniformis*. This provides strong evidence that Eurasian badgers are parasitized by a hookworm species, *U. criniformis*, that is molecularly distinct from *U. stenocephala*.

Numerous authors have attempted to identify consistent morphological differences between these *U. stenocephala* and *U. criniformis*, but results have often been inconclusive, especially when host species was used as a key differentiating factor (Cameron, 1924; Fülleborn, 1924; Ransom, 1924). If Eurasian badgers can host both species as supported by Górski et al (2023), then host-based assumptions may have confounded earlier analyses. It would be prudent to assess the prevalence of *U. stenocephala* in badgers using molecular tools such as ITS rDNA or *cox*1 markers. Host species should no longer be used to determine hookworm identity, as hookworms can act as generalists, infecting and completing their life cycles in a wide range of hosts. Therefore, host association is not a reliable indicator of species identity.Our DNA sequence comparisons revealed a high degree of similarity (>99% identity) between *U. criniformis* and sequences annotated as *Arthrostoma* sp. from the Asian badger (*Meles leucurus*) in China. Specifically, the *cox*1 sequences (MW517831 and MW517832) are reference sequences for *Arthrostoma leucurus*, recently described by Liu et al (2022). This high-sequence identity strongly suggests that these hookworms are conspecific. This is not surprising, given the close evolutionary relationship between *M. meles* and *M. leucurus* (Kinoshita et al., 2017). The distribution boundary between these 2 badger species lies near the Volga River in Russia, with *M. meles* to the west and *M. leucurus* to the east. It is therefore plausible that the hookworm species parasitized a common ancestral badger species prior to their divergence.

The morphology of the buccal capsule in *A. leucurus* led Liu et al (2022) to classify it within the genus *Arthrostoma*, which is characterized by the presence of articulated buccal plates (Cameron, 1927; Liu et al., 2022). These plates, typically 8 to 10 in number, may not always be fully articulated (Cameron, 1927; Liu et al., 2022). Comparing the published morphology of *A. leucurus* with *U. criniformis* as described by Ransom (1924) reveals similarities, particularly in the male bursa. Liu et al (2022) described the medio-lateral ray as slightly thicker than the postero-lateral ray and much thicker than the externo-lateral ray, so consistent with Ransom’s (1924) description of *U. criniformis*. While our specimens matched Ransom’s description and showed similar buccal capsule morphology, we could not confirm the presence of articulated plates without direct examination of *A. leucurus* material. Cameron (1924) provided a detailed revision of *U. criniformis* and *U. stenocephala*, concluding they were the same species and proposing the new genus *Dochmoides*. Cameron (1927) introduced the genus *Arthrostoma* without linking it to *Dochmoides* (=*Uncinaria*). Based on our findings, we interpret that hookworms in *Meles* species are conspecific. Therefore, *A. leucurus* should be considered a junior synonym of *U. criniformis* unless future evidence demonstrates distinct species in Eurasian and Asian badgers.

Our mitogenome-based phylogenetic analysis revealed that the genus *Uncinaria* is polyphyletic. Currently, the only other *Uncinaria* species with a sequenced mitogenome is *U. sanguinis*, a hookworm of sea lions (Haynes et al., 2014; Marcus et al., 2014). To better resolve hookworm evolutionary relationships, further mitogenomic characterization is needed for additional *Uncinaria* species, particularly *U. stenocephala*, as well as for related genera such as *Globocephalus, Hypodontus, Agriostomum, Galoncus, Arthrocephalus, Placoconus, Tetragomphius* and *Arthrosoma* (Ilik et al., 2024). Ideally, the type species of each of these genera should be reviewed and their mitogenomes sequenced, including that of the canine hookworm *U. stenocephala*, to clarify their phylogenetic relationships.

In this study, we characterized *U. criniformis* from the Eurasian badger and generated reference sequence data for 2 key molecular markers: the complete ITS rDNA region and the mitochondrial genome. We employed genome skimming via NGS as a cost-effective method to recover these markers, enabling robust species identification. The ITS rDNA region is widely used in molecular surveys, such as nemabiome or amplicon-based metabarcoding, to profile nematode communities in samples like faeces (Stocker et al., (2023); Abdullah et al., (2025) (Avramenko et al., 2015; Redman et al., 2019). Our demonstration that *U. criniformis* is molecularly distinct from *U. stenocephala* provides a reliable basis for distinguishing these species in future parasitological surveys.

## Data Availability

The vouchers of *U. criniformis* have been deposited at the IPCAS Institute of Parasitology, Academy of Sciences of the Czech Republic, České Budějovice, Czech Republic under IPCAS N-1292. Sequence data were deposited in GenBank under the following accession numbers: PV763276-PV763277 and PV766792- PV766793. Raw FastQ sequence data were deposited at SRA NCBI BioProject: PRJNA1273505. Intermediate data for the samples and analyses are available at LabArchives: https://dx.doi.org/10.25833/yap8-7191.
